# Brazos Valley Medical Association

**Published:** 1904-08

**Authors:** W. A. Briggs

**Affiliations:** Secretary


Brazos Valley Medical Association.—The Brazos Valley Medical Association held its seventeenth semi-annual meeting at Caldwell on May 10th and 11th. The following papers were read: "Autotoxemia or Malaria," Dr. Ben. H. Brodnax, Brodnax, La.; "Peculiar Gin Accident," Dr. J. P. Oliver, Caldwell, Texas; "Rheumatoid Arthritis," Dr. J. W. Torbett, Marlin, Texas; "A Rapid Method of End to End Anastomosis," Dr. W. W. Greer, Cameron, Texas; "Two Cases of Injury to Spinal Column," Dr. J. E. Thompson, Gaveston, Texas; "New Drugs and Procedures in Ocular Therapeutics," Dr. Joseph Mullen, Houston, Texas.

Dr. Thompson brought with him and exhibited a beautiful dissection showing the various methods in operations for hernia.

The following officers were elected: President, Dr. E. N. Shaw, Cameron; first vice-president, Dr. A. G. Krueger, Caldwell; second vice-president, Dr. H. E. Baine, Lexington; secretary and treasurer, Dr. W. B. Briggs, Easterly.

The secretary was instructed to send accounts to delinquents. If not settled in thirty days, drafts were to be drawn on them through the banks. If these were not paid before the next meeting, the names of all delinquents were to be read out before the association on the second Tuesday in November, prox., as suspended.

Due notice will be given of the next place of meeting.

Members of the association are earnestly requested to prepare papers for the next meeting. Please send subjects to Dr. W. B. Briggs at Easterly, not later than September 30th, as the program is desired at least one month before the time of meeting. Do not pass this matter idly by. There is ample time for preparation. A few papers from reputable physicians outside of the membership will be received. W. A. Briggs, Secretary.

